# Single-cell and bulk RNA sequencing reveal heterogeneity and diagnostic markers in papillary thyroid carcinoma lymph-node metastasis

**DOI:** 10.1007/s40618-023-02262-6

**Published:** 2023-12-26

**Authors:** D.-N. Lu, W.-C. Zhang, Y.-Z. Lin, H.-Y. Jiang, R. He, S.-L. Li, Y.-N. Zhang, C.-Y. Shao, C.-M. Zheng, J.-J. Xu, M.-H. Ge

**Affiliations:** 1 Otolaryngology & Head and Neck Center, Cancer Center, Department of Head and Neck Surgery, Zhejiang Provincial People’s Hospital (Affiliated People’s Hospital, Hangzhou Medical College), Hangzhou, 310014 Zhejiang People’s Republic of China; 2 Second Clinical Medical College, Zhejiang Chinese Medical University, Hangzhou, China; 3 Key Laboratory of Endocrine Gland Diseases of Zhejiang Province, Hangzhou, 310014 Zhejiang People’s Republic of China; 4 School of Basic Medical Sciences and Forensic Medicine, Hangzhou Medical College, Hangzhou, 310059 China; 5 Clinical Research Center for Cancer of Zhejiang Province, Hangzhou, 310014 Zhejiang People’s Republic of China

**Keywords:** Papillary thyroid carcinoma, Lymph-node metastasis, Single-cell sequencing, Diagnostic model

## Abstract

**Purpose:**

Papillary thyroid carcinoma (PTC) is characterized by lymph-node metastasis (LNM), which affects recurrence and prognosis. This study analyzed PTC LNM by single-cell RNA sequencing (scRNA-seq) data and bulk RNA sequencing (RNA-seq) to find diagnostic markers and therapeutic targets.

**Methods:**

ScRNA-seq data were clustered and malignant cells were identified. Differentially expressed genes (DEGs) were identified in malignant cells of scRNA-seq and bulk RNA-seq, respectively. PTC LNM diagnostic model was constructed based on intersecting DEGs using glmnet package. Next, PTC samples from 66 patients were used to validate the two most significant genes in the diagnostic model, S100A2 and type 2 deiodinase (DIO2) by quantitative reverse transcription-polymerase chain reaction (RT-qPCR) and immunohistochemical (IHC). Further, the inhibitory effect of DIO2 on PTC cells was verified by cell biology behavior, western blot, cell cycle analysis, 5-ethynyl-2′-deoxyuridine (EdU) assay, and xenograft tumors.

**Results:**

Heterogeneity of PTC LNM was demonstrated by Kyoto Encyclopedia of Genes and Genomes (KEGG) and Gene Ontology (GO) analysis. A total of 19 differential genes were used to construct the diagnostic model. S100A2 and DIO2 differ significantly at the RNA (*p* < 0.01) and protein level in LNM patient tissues (*p* < 0.001). And differed in PTC tissues with different pathologic typing (*p* < 0.001). Further, EdU (*p* < 0.001) and cell biology behavior revealed that PTC cells overexpressed DIO2 had reduced proliferative capacity. Cell cycle proteins were reduced and cells are more likely to be stuck in G2/M phase (*p* < 0.001).

**Conclusions:**

This study explored the heterogeneity of PTC LNM using scRNA-seq. By combining with bulk RNA-seq data, diagnostic markers were explored and the model was established. Clinical diagnostic efficacy of S100A2 and DIO2 was validated and the treatment potential of DIO2 was discovered.

**Supplementary Information:**

The online version contains supplementary material available at 10.1007/s40618-023-02262-6.

## Introduction

Thyroid carcinoma (TC) is a type of malignant cancer whose incidence has increased considerably in recent years, mainly due to improved diagnostic techniques and an increase in the frequency of routine medical checkups [1, 2]. The main pathological type of TC is PTC—considered inert—with a low mortality rate and a good prognosis [3]. However, LNM occurs in 40–90% of patients with PTC and affects local recurrence and prognosis, making LNM an important factor to consider when treating TC [4].

The development of RNA sequencing and microarray technology has allowed the analysis of DEGs to improve our understanding of PTC [5, 6]. Traditional bulk RNA-seq data provide the average expression levels from a diverse set of cells, which can be used to explore differences between different tissue types; however, these data cannot be used to analyze specific cell types [7]. ScRNA-seq allows the unbiased genome-wide analysis of the transcriptomes of many individual cells, which can help to characterize the cellular heterogeneity within samples [8, 9]. Yet, bulk RNA-seq still has advantages of economy and practicality especially in daily clinical work. In this study, we analyzed functional heterogeneity and discovered diagnostic markers of PTC LNM by combining scRNA-seq and bulk RNA-seq data, which balanced between accuracy and feasibility.

S100A2 is an important member of the S100 protein family and abnormal S100A2 expression has been reported to affect multiple cellular functions, including calcium homeostasis, enzyme activity, and protein phosphorylation [10]. In addition, S100A2 has been associated with malignant progression in pancreatic, colorectal, lung, and gastric cancers [11–15]. DIO2 is a member of the iodothyronine deiodinase family [16]. Many studies have demonstrated that DIO2 plays an important role in advanced tumors including prostate cancer [17], squamous cell carcinoma tumor [18], and mesothelioma [19]. Recent studies have also shown that DIO2 is under expressed in almost all PTC cases [20]. Unlike other studies that explored differences between tumors and situ tissue, this study is focus on a single biological feature of tumors—LNM.

## Methods

### All experiments were repeated more than three times

#### Data acquisition

scRNA-seq data (GSE184362) were downloaded from the Gene Expression Omnibus (GEO; https://www.ncbi.nlm.nih.gov/geo/query/acc.cgi). The Cancer Genome Atlas (TCGA)-thyroid carcinoma bulk RNA-seq and clinical data were downloaded from the Genomic Data Commons data portal (https://portal.gdc.cancer.gov/).

#### scRNA-seq data preprocessing

The gene expression patterns of each cell were detected using the DropletUtils [21] R package. Barcodes that were not expressed by any cells were filtered out, and data were further filtered according to the number of unique molecular identifiers in each cell. Gene expression was thereafter determined using the Scater [22] package. Cells with a mitochondrial gene expression ratio > 10% and ribosomal gene expression ratio < 10% were filtered out. Finally, the expression matrix of each filtered sample was normalized using the NormalizeData function in Seurat [23].

#### Principal component analysis (PCA)

The top 2000 genes with the most obvious differences in expression between cells were screened using FindVariableFeatures in Seurat to highlight biological signals in single-cell datasets. Expression data were then scaled linearly using ScaleData in Seurat and PCA analysis was performed using the RunPCA function.

#### Cell clustering and annotation

Having selected the principal component with the largest standard deviation, cell clustering analysis was performed using the FindNeighbors and FindClusters functions in Seurat. Uniform Manifold Approximation and Projection (UMAP) analysis was then performed using the RunUMAP package in Seurat.

#### Marker gene identification

DEGs between each cluster and all other cells were identified using the FindMarkers function in Seurat (log2FC ≥ 0.1, minimum expression ratio = 0.25, *p* ≤ 0.05). Marker genes (top 500 logFC values) were used to label cells and generate a cluster diagram [24].

#### Malignant cell identification

To identify malignant cells with abnormal gene expression according to the position of each gene on the chromosome, the inferCNV package was used with the following parameters: "denoise", default Hidden Markov Model settings, "cutoff" = 0.1. Using immune cells as a reference and thyrocytes as candidate cells, copy-number variation (CNV) was calculated to identify malignant and non-malignant thyroid cells. To reduce false-positive calls, a default Bayesian latent mixture model was used to determine the posterior probability of CNV changes in each cell, with a default threshold of 0.5.

#### CIBERSORTX and CIBERSORT analysis

An scRNA-seq data signature was constructed according to the expression levels in each cell type using the CIBERSORTX (https://cibersortx.stanford.edu/) online tool. The proportions of cell types in each sample were calculated using CIBERSORT [25]. Differences in the proportion of each cell type between groups were then calculated.

#### Differential expression analysis

The FindMarkers function based on wilcox.test in Seurat (for scRNA-seq) and the edgeR [26] package (for bulk RNA-seq) were used for differential expression analysis. To ensure the effect of the diagnostic model, we set the FC at 1.2 to better characterize the heterogeneity of the PTC LNM.: minimum expression ratio = 0.25, *p* < 0.05, and fold change > 1.2.

#### Gene enrichment analysis

Functional enrichment analysis of candidate genes was performed using the GO [27] and KEGG pathway [28] databases. Fisher's exact tests were used to identify which genes were most associated with specific functions. A smaller *p* value indicated more significant enrichment.

#### Cell–cell interaction network analysis

CellChat v1.1 [29] was used to infer cell-to-cell communication based on receptor–ligand gene expression values for each cell type. Intercellular receptor–ligand pairs were then obtained to determine relationship networks. Cell–cell communication analysis was performed using the “CellchatDB.human” ligand–receptor interaction database with default settings. The total number and intensity of interactions were compared by merging the CellChat objects for each group using the mergeCellChat function. Differences in the number or strength of interactions between different cell populations were visualized using the netVisualDiffInteraction function. Differentially expressed signaling pathways were identified using the rankNet function and the communication network was visualized using the plotGeneExpression function.

#### Transcription factor regulation analysis

Regulators in scRNA-seq data were identified using SCENIC [30] with human hg38-500bp_up_and_100bp_down_tss data. The regulatory activities of corresponding transcription factors (TFs) were inferred and used to construct a TF regulatory network. The GENIE3 package was used to deduce co-expression modules between TFs and candidate target genes. Cis-regulatory motifs for each co-expression module were analyzed using the RcisTarget package to construct a gene regulatory network module containing TFs and target genes. Finally, regulator activity was analyzed using AUCell software and area under the curve (AUC) values were calculated for each gene regulatory network module to assess the activation of gene regulatory network modules in cells.

#### Diagnostic model construction

Using the binomial method in the glmnet package, a diagnostic model (lambda.min = 0.0252) was constructed based on TCGA–THCA bulk RNA-seq data. Model reliability was verified by receiver-operating characteristic analysis between model predictions and actual analysis using the pROC package. The risk score was calculated as follows:

$${\text{Risk}} = \exp \left( {{\text{expression 1}} \times {\text{coefficient 1}} + {\text{expression 2}} \times {\text{coefficient 2}} + {\text{expression 3}} \times {\text{coefficient 3}} + \cdots } \right),$$

where “expression” refers to gene expression levels and “coefficient” refers to the coefficient corresponding to each gene (Table 1).

**Table 1:** Genes in the diagnostic model

Variable	Coefficient
(Intercept)	5.110810415
*DIO2*	0.156115542
*ID4*	0.055988881
*HSPA5*	0.128243988
*LINC01315*	0.209007783
*PAX8*	0.212704648
*PDE8B*	− 0.217710385
*SLC25A29*	− 0.022502554
*CLIC3*	− 0.000196994
*LPCAT2*	0.349791278
*S100A2*	− 0.103768403
*RPS4Y1*	− 0.026629823
*S100A5*	− 0.040491598
*ZFP36L1*	− 0.138467204
*CCDC80*	− 0.001329735
*YBX3*	− 0.16833279
*PTPRF*	0.100690014
*NPC2*	− 0.21793595
*DNAJC21*	− 0.812103548
*SNX1*	− 0.270370509

#### The tumor immune dysfunction and exclusion (TIDE) score

According to gene expression levels, each sample was scored using the TIDE website (http://tide.dfci.harvard.edu/login/) to predict the response to immune checkpoint blockade. Wilcoxon tests were then used to compare TIDE scores between groups.

#### Immune infiltration analysis

Based on gene expression data, each sample was scored for immune infiltration using CIBERSORT. Correlations between the scores for 22 immune cell types were then calculated and differences in immune scores were compared between groups.

#### Clinical samples

Tissue samples (*n* = 66) were collected from patients with PTC at the Zhejiang Provincial People’s Hospital, Hangzhou, Zhejiang Province, China, and stored at − 80 °C for subsequent experiments (Table 2). The study was approved by the Ethics Committee of The Zhejiang Provincial People’s Hospital (QT2022410). Written informed consent was obtained from all patients.

**Table 2:** Clinicopathological data for samples from Zhejiang Province People's Hospital

Patient ID	Sex	Age	Tumor size (cm)	TNM stage	Clinical stage	Histologic subtype	Concomitant Hashimoto’s thyroiditis	Extra- thyroid extension	Vascular invasion	FT3 (2.14–4.21 pmol/L)	FT4 (5.90–12.50 pmol/L)	TSH (0.34–5.60 mIU/L)	TGAb (0.00–4.00 IU/mL)	TPOAb (0.00–9.00 IU/mL)
1	F	40	0.9	T1bN1aM0	1	Classical	Yes	Yes	No	3.23	7.56	2.13	597.00	348.00
2	M	25	3.2	T2N1aM0	1	Classical	No	Yes	Yes	3.90	8.67	2.86	< 0.90	< 0.40
3	F	30	1.8	T1bN1bM0	1	Classical	No	No	No	3.32	9.46	2.40	9.60	7.20
4	F	32	0.5	T1aN0M0	1	Classical	No	No	No	3.09	8.78	1.59	22.40	2.20
5	F	46	1.1	T1bN0M0	1	Classical	No	Yes	No	4.11	11.75	1.13	< 0.90	< 0.40
6	M	56	1.6	T1bN1bM0	2	Classical	No	No	Yes	3.61	10.56	2.36	< 0.90	2.70
7	F	41	0.5	T2N0M0	1	Follicular	No	No	No	3.01	10.11	1.08	19.60	1.10
8	M	68	1.8	T1bN1bM0	2	Classical	No	Yes	No	2.82	8.80	0.99	< 0.90	1.40
9	F	22	2.5	T2N1bM0	1	Classical	Yes	Yes	Yes	3.20	8.28	3.76	< 0.90	125.00
10	M	54	1.3	T4N1bM0	1	Classical	No	Yes	No	3.55	9.55	1.07	< 0.90	< 0.40
11	F	59	0.8	T1aN0M0	1	Classical	No	No	No	2.65	9.62	2.64	124.10	0.70
12	M	46	2.6	T2N1bM0	1	Classical	No	No	Yes	3.62	8.90	3.28	< 0.90	1.90
13	M	48	1	T1aN0M0	1	Classical	Yes	No	No	3.40	9.04	1.36	< 0.90	25.90
14	M	50	0.9	T1aN0M0	1	Classical	No	Yes	No	3.66	8.68	1.77	< 0.90	< 0.40
15	F	43	1	T1aN0M0	1	Follicular	No	No	No	3.95	8.78	3.09	< 0.90	6.60
16	M	49	1.2	T1bN0M0	1	Classical	No	No	No	3.75	7.08	1.84	< 0.90	< 0.40
17	F	46	0.7	T1aN0M0	1	Classical	No	Yes	No	3.76	8.79	2.28	< 0.90	1.50
18	F	55	1.1	T1bN1aM0	2	Classical	No	Yes	No	3.29	11.34	1.70	2.70	0.50
19	M	32	2.8	T2N1aM0	1	Classical	No	No	No	3.83	12.04	2.52	< 0.90	0.70
20	M	36	1.5	T1bN1bM0	1	Tall cell	No	Yes	No	3.82	11.17	1.13	< 0.90	1.00
21	F	32	0.9	T1aN0M0	1	Classical	No	Yes	No	3.52	8.37	2.56	16.50	8.60
22	F	52	0.9	T1bN0M0	1	Classical	No	Yes	No	3.56	9.20	3.15	< 0.90	0.70
23	F	33	1.2	T1bN0M0	1	Classical	No	Yes	No	3.56	6.97	2.64	< 0.90	< 0.40
24	F	34	2.5	T2N1bM0	1	Classical	Yes	Yes	Yes	3.17	7.66	3.31	458.30	39.70
25	F	51	1.3	T1bN1bM0	1	Tall cell	No	Yes	Yes	3.70	7.15	2.71	< 0.90	0.60
26	F	29	1.4	T1bN1bM0	1	Classical	Yes	Yes	Yes	4.20	9.47	1.76	117.60	228.20
27	F	34	2.3	T2N0M0	1	Classical	Yes	Yes	No	3.09	10.08	2.85	65.80	7.80
28	M	65	0.7	T1aN0M0	1	Classical	No	Yes	No	3.19	8.56	1.17	< 0.90	< 0.40
29	F	37	2	T1bN0M0	1	Classical	No	Yes	Yes	3.33	7.69	1.42	< 0.90	0.80
30	M	38	1.8	T1bN1bM0	1	Classical	Yes	Yes	No	3.16	8.55	1.89	51.40	0.80
31	M	64	2.2	T2N1aM0	2	Classical	No	Yes	Yes	2.30	8.40	1.74	< 0.90	1.40
32	F	48	1.2	T1bN1aM0	1	Classical	No	Yes	Yes	3.59	8.15	1.61	< 0.90	0.50
33	M	52	1.2	T1bN0M0	1	Follicular	No	No	No	3.31	7.16	3.32	< 0.90	2.90
34	F	70	2	T1bN1aM0	2	Classical	No	No	No	2.75	9.64	3.80	< 0.90	1.70
35	F	52	1.5	T1bN1aM0	1	Classical	No	No	No	NA	NA	NA	NA	NA
36	M	39	2.3	T2N1bM0	1	Classical	Yes	Yes	No	4.13	11.08	8.36	9.40	48.00
37	F	46	2.1	T2N1aM0	1	Classical	No	No	No	3.60	11.36	1.73	< 0.90	0.60
38	M	58	1.9	T2N1bM0	2	Classical	No	Yes	No	3.29	8.46	2.68	< 0.90	< 0.40
39	F	65	3.1	T3N0M0	2	Classical	No	No	No	3.67	9.57	2.57	< 0.90	2.30
40	F	45	1.7	T1bN0M0	1	Classical	No	Yes	No	3.46	8.58	3.05	1.90	1.90
41	F	53	1.4	T1bN0M0	1	Classical	Yes	Yes	No	3.12	8.74	1.55	< 0.90	162.30
42	M	27	3.4	T4aN1bM0	1	Classical	No	Yes	No	3.69	7.92	1.87	< 0.90	< 0.40
43	F	53	2.1	T2N1bM0	1	Classical	Yes	No	No	NA	NA	NA	NA	NA
44	F	44	0.7	T1aN0M0	1	Follicular	Yes	Yes	No	3.66	8.02	3.51	< 0.90	405.50
45	F	29	0.7	T1aN0M0	1	Classical	Yes	No	No	3.33	8.23	4.53	1736.90	221.60
46	F	45	0.4	T1aN0M0	1	Follicular	No	No	No	3.66	8.39	2.24	< 0.90	< 0.40
47	F	23	4.4	T4N1bM0	1	Tall cell	No	Yes	Yes	3.94	10.29	1.48	< 0.90	3.20
48	F	53	1.4	T1bN0M0	1	Classical	No	No	No	3.44	9.09	1.03	< 0.90	0.60
49	F	41	1.3	T1bN0M0	1	Classical	No	Yes	No	2.61	9.31	3.63	7.80	26.30
50	F	22	4.5	T4N1bM0	1	Classical	Yes	Yes	Yes	4.30	11.00	1.57	< 0.90	58.70
51	F	64	3.2	T2N0M0	1	Classical	No	No	No	3.27	8.98	1.73	< 0.90	< 0.40
52	F	33	1.2	T1bN0M0	1	Classical	No	Yes	No	3.74	7.05	2.56	< 0.90	< 0.40
53	M	84	3.1	T4aN1bM0	3	Classical	No	Yes	Yes	3.65	9.52	1.65	< 0.90	< 0.40
54	F	35	1.7	T1bN1aM0	1	Classical	No	Yes	No	3.26	7.76	4.48	< 0.90	< 0.40
55	F	68	3	T2N1bM0	2	Classical	No	No	Yes	3.67	10.06	1.11	< 0.90	1.10
56	M	55	0.6	T1bN1aM0	1	Classical	No	No	No	3.35	7.17	1.23	< 0.90	0.60
57	M	49	3.8	T3aN1bM0	1	Classical	No	No	Yes	3.43	8.46	3.49	< 0.90	1.20
58	F	72	2.5	T2N1bM0	2	Classical	No	No	No	2.45	8.34	0.58	< 0.90	3.00
59	F	38	2.7	T2N1bM0	1	Classical	Yes	No	Yes	3.52	7.48	1.93	3.90	353.10
60	F	22	2.3	T2N1aM0	1	Tall cell	No	Yes	No	3.15	11.01	4.38	< 0.90	1.40
61	F	50	1.8	T1bN1bM0	1	Classical	No	Yes	Yes	2.94	7.34	2.99	16.70	1.60
62	F	59	1.4	T1bN1bM0	2	Classical	No	Yes	No	3.15	9.78	2.37	< 0.90	0.70
63	F	50	2.3	T2N0M0	1	Follicular	Yes	No	No	2.80	8.20	2.00	1.10	633.80
64	F	16	2.7	T2N1bM0	1	Classical	Yes	No	No	3.90	6.47	21.29	178.00	> 1000.00
65	F	46	2.5	T2N1aM0	1	Classical	No	No	No	3.60	11.36	1.73	< 0.90	0.60
66	M	70	2.9	T2bN1M0	2	Classical	No	Yes	No	3.50	8.32	0.23	< 0.90	1.30

#### RT-qPCR

Total RNA was obtained from cells and frozen samples using a TRIzol kit (Invitrogen, California, USA). RNA purity and concentration were determined using an Ultraviolet spectrophotometer (Thermo Fisher Scientific, Massachusetts, USA) after 5 µL RNA samples had been diluted 20-fold with RNase-free ultrapure water. RNA transcripts were then reverse transcribed into cDNA using a Prime Script RT Master Mix kit (Beyotime, Shanghai, China) and RT-qPCR analysis was performed using SYBR Premix Ex Taq™ II (Takara Bio, Tokyo, Japan) with GAPDH and U6 as endogenous controls. The following thermal cycling conditions were used: 94 °C for 4 min, 95 °C for 1 min, 40 cycles, 60 °C for 1 min, and 70 °C for 1 min. All experiments were repeated at least three times. The following primers were used: *DIO2*, forward 5′-TCCTGGCTCTCTATGACTCGG-3′ and reverse 5′-TAC TGGAGACATGCACCACAC-3′; *GAPDH*, forward 5′-CTGGGCTACACTGAGCACC-3′ and reverse 5′-AAGTGGTCGTTGAGGGCAATG-3′; *S100A2*, forward 5′-GCGACAAGTTCAAGC TGAGTAAG-3′ and reverse 5′-GACAGTGATGAGTGCCAGGAAA-3′. All values were normalized to *GAPDH* and the fold change was quantified using the 2 − ΔCt method.

#### Immunohistochemical analysis

After tissue sections had been deparaffinized and rehydrated, antigen retrieval was performed using 0.01 M citric acid buffer. The sections were then washed with phosphate buffer solution, blocked using normal serum, and incubated overnight with anti-DIO2 (abcam, Massachusetts, USA) and anti-S100A2 (abcam, Massachusetts, USA) antibodies at 4 °C, followed by secondary antibodies at 37 °C for 30 min. After staining with diaminobezidin solution, sections were observed under a microscope (Olympus, Tokyo, Japan).

#### Cell culture

BCPAP and IHH4 cells (PTC cell lines) obtained from the National Collection of Authenticated Cell Cultures (Beijing, China) were cultured in Roswell Park Memorial Institute (RPMI)-1640 medium (Thermo Fisher Scientific) containing 10% fetal bovine serum (FBS; Gibco, California, USA) in a humidified 5% CO_2_ atmosphere at 37 °C.

#### Cell stable transfection

Cells were seeded in a 6-well plate at a density of 1.0–1.5 × 10^5^ cells per well in an antibiotic-free RPMI-1640 medium. Virus (GeneChem, Shanghai, China) with puromycin resistance and two paired transfection reagents (Three wells each) were added in each well separately after 24 h according to the manufacturer’s protocol. The medium was changed after 12–18 h and screened with puromycin for 3 days. The wells with the best overexpression results were selected for incubation.

#### CCK-8 assay

Cell viability was assessed using a cell counting kit-8 (Jiancheng, Nanjing, China). Transfected cells were seeded into 96-well plates and incubated at 37 °C for 6, 12, 24, 48, and 72 h. Subsequently, 10 μL of CCK-8 solution was added and the cells allowed to incubate for 1 h. Absorbance was determined using a microplate reader (Bio-Rad, Hercules, California, USA) at 450 nm.

#### Transwell assay

Cell invasion and migration were examined using Transwell chambers (8 µm pore size; Corning, New York, USA) pre-coated with and without Matrigel, respectively. Briefly, cell suspensions (1–2 × 10^5^ cells/mL) were prepared using a serum-free culture RPMI-1640 medium and added to the upper chamber (200 µL), while 500 µL RPIM-1640 supplemented with 10% FBS was added to the lower chamber. After incubation at 37 °C with 5% CO_2_ for 12 h to assess migration or for 24 h to assess invasion, the top surface of the membrane was wiped with a cotton swab. Cells on the bottom surface were fixed with 4% formaldehyde for 10 min, stained with 0.1% crystal violet, and imaged using a microscope (Nikon, Tokyo, Japan).

#### Wound-healing analysis

Transfected cells were seeded in a 12-well plate and cultured at 37 °C until they reached 85–90% confluence. A straight wound was made in the middle and 1/3 of the sides of the plate with the same force using a sterile 10 µL pipette tip, after which the medium was aspirated and replaced with serum-free RPMI-1640. Cell migration was observed under a microscope (Nikon, Tokyo, Japan) and imaged immediately (0 h) and after incubation at 37 °C for 12 h.

#### Colony formation assay

Transfected cells were seeded in 6-well plates and incubated at 37 °C with 5% CO_2_ for 1 week. The cells were then immobilized with 4% paraformaldehyde fix solution, stained with crystal violet, and counted using ImageJ software, National Institutes of Health, USA.

#### Cell cycle analysis

The cell cycle was analyzed using the Cell Cycle Kit (Liankebio, China) following the manufacturer’s instructions. The PTC cell line (IHH4 and BCPAP) and its DIO2 overexpressed cells (2.5 × 10^5^ or 2.0 × 10^5^ cells/well) were seeded in 6-well plates. After 24 h of incubation at 37 ◦C, the cells were collected, washed with PBS, and stained in 1 mL DNA staining solution containing 10 µl permeabilization solution, at room temperature, and in the dark for 30 min. For analysis of the cell cycle, the lowest sampling speed was subjected to Navios flow cytometry (Beckman-Coulter, USA). The results were analyzed using Flowjo.

#### Western blotting

After successful transfection, the cells were harvested and lysed on ice for 10 min using western and IP lysis buffer (# P0013, Beyotime Institute of Biotechnology, China) containing PMSF. Total protein concentration was determined using a bicinchoninic acid protein assay kit (Thermo Fisher Scientifc, USA). The protein samples were resolved through SDS-PAGE precast Tris-Gly gels (4–20%, # P0524M, Beyotime Institute of Biotechnology, China), and then transferred onto PVDF membranes. The membranes were blocked with TBST with 1% Tween-20 containing 5% skim milk for 1 h. This was followed by incubation with corresponding primary antibodies at 4^◦^C overnight, and then with suitable secondary antibodies conjugated with HRP at the room temperature for 60 min. The membranes were analyzed by FDbioDura ECL Kit (#FD8020, Fdbio Science, China) and imaged with the ChemiDoc-MP imager (Bio-Rad, USA). Band density was quantified by ImageJ software.

#### 5-Ethynyl-2-deoxyuridine (EdU) incorporation assay

After transfection, cells were seeded in 96-well plate in triplicate. Then, the medium was replaced with fresh medium containing EdU (Riobio, Guangzhou, China). After culturing for 2 h, EdU detection was also performed according to the protocol using the kFluor555 Click-iT EdU Cell Proliferation Kit (Biotech, Nanjing, China) according to the manufacturer’s instructions. Images were taken by a Leica DMi8 Microscope (Leica, Germany), and the EdU-positive cells were counted.

#### Xenograft tumors

Female BALB/c nude mice (age: 4–6 weeks) were purchased from Shanghai SLAC Laboratory Animal Co. Ltd. (Shanghai, China). IHH4 cells (1 × 10^6^ cells) with stable overexpression of DIO2 and control IHH4 cells were injected subcutaneously into the backs of the mice. The long diameter and short diameter of the tumors were assessed every 2 days. The observation period lasted for 3 weeks. At the end of this in vivo study, the mice were sacrificed, and the tumors were removed and weighed. The animal study was approved by the Ethics Committee of The Zhejiang Provincial People’s Hospital and was conducted in accordance with the Guide for the Care and Use of Laboratory Animals.

#### Statistical analysis

All statistical analyses were performed using the R Statistical Environment v.4.0.1 (R Foundation for Statistical Computing, Vienna, Austria) and GraphPad Prism v.8.01 (GraphPad Software, California, USA). Student’s *t* tests were used to compare data between the test and control groups; *p* values < 0.05 were considered significant.

## Results

### Establishment of a single-cell atlas for PTC

**Fig. 1:**
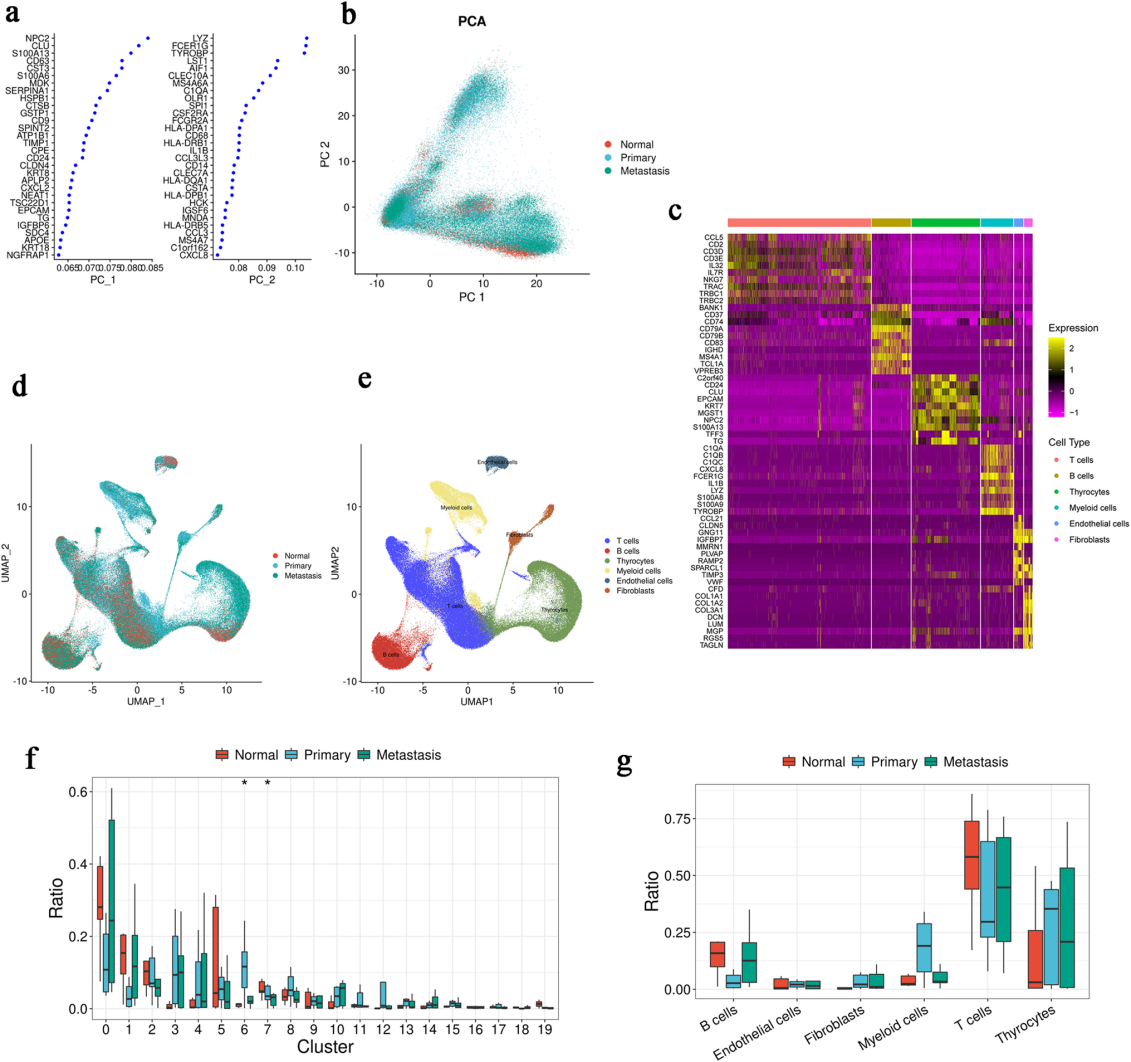
The establishment of single-cell atlas for PTC LNM. **a** Top two PC gene contributions in scRNA-seq data. **b** PCA groupings or scRNA-seq data. **c** Expression heatmap of the top ten marker genes in various cells. **d**, **e** Cell grouping and type clustering through UMAP. **g** Cell proportion analysis in samples. **h** Cell proportion analysis in cell types. *p < 0.05

A total of 187,707 cells were collected from seven patients for the Primary group and eight metastatic lymph-node tissues from seven patients for the Metastasis group. After quality control filtering, 185,006 cells were retained for PCA after linear scaling. We scaled the cells in two dimensions to better show the location of the cells (Fig. 1a, b). All cells were identified by the genes that characterize each types (Fig. 1c), and cell clustering was accomplished (Fig. 1d, e). Next, we determined the proportion of each cell type in each sample (Fig. 1f) and found no significant difference in the proportions of different cell types (Fig. 1g). Therefore, it is necessary to further explore the heterogeneity of PTC LNM.

### Functional heterogeneity of malignant cells

**Fig. 2:**
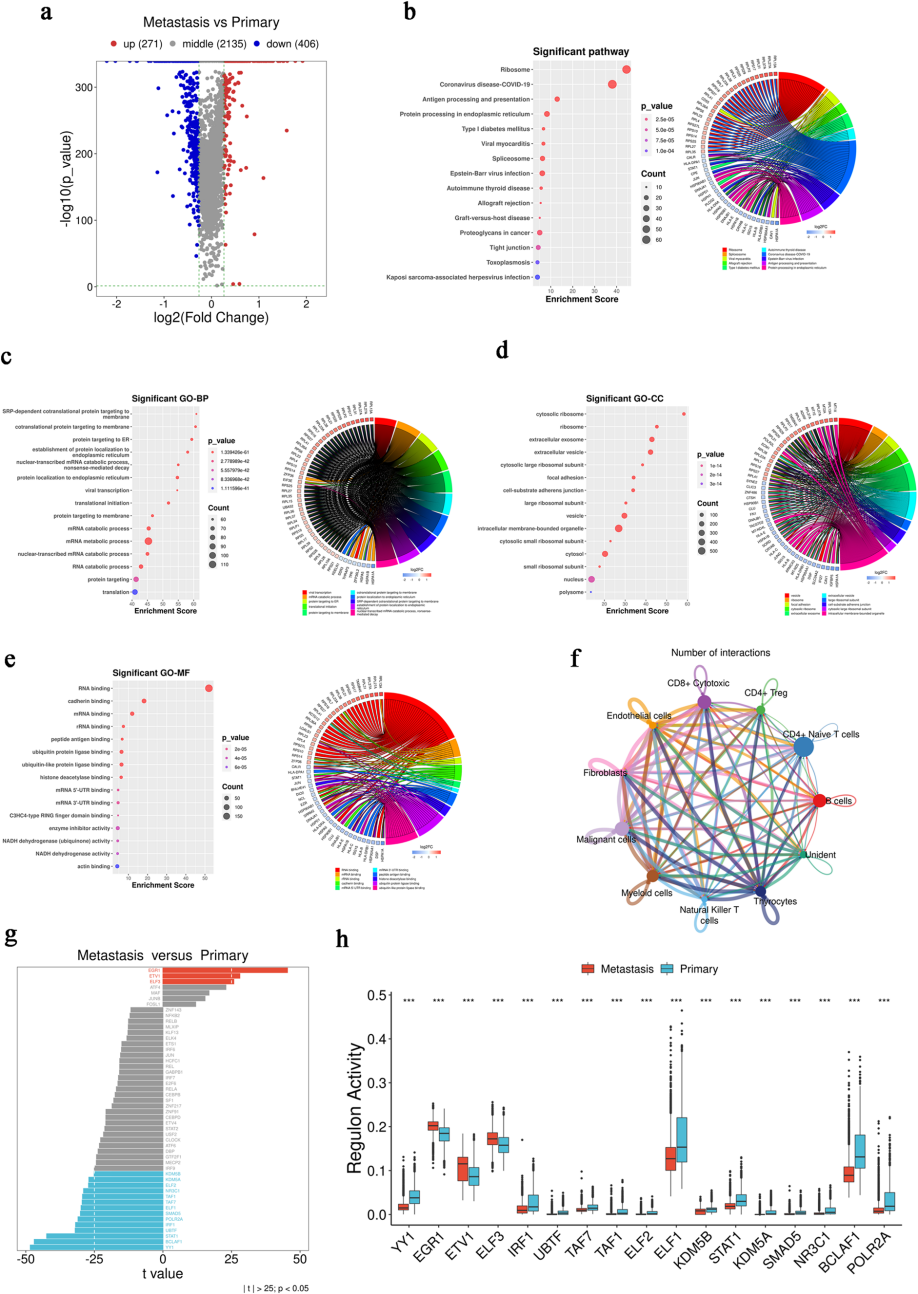
Mechanism of LNM in PTC.** a** Volcano map of DEGs between groups in bulk RNA-seq data. **b** KEGG analysis of DEGs in bulk RNA-seq data and circle plot of the top ten enriched KEGG pathways corresponding to the first (maximum) 50 genes based on absolute log_2_FC values. **c**–**e** GO analysis of BP, CC, and MF for DEGs in bulk RNA-seq data and circle plot of the top ten enriched GO–BP, GO–CC, and GO–MF pathways corresponding to the first (maximum) 50 genes based on absolute log_2_FC values. **f** Between-group differences in the proportion of each cell type in bulk RNA-seq data, ***p < 0.001

**Fig. 3:**
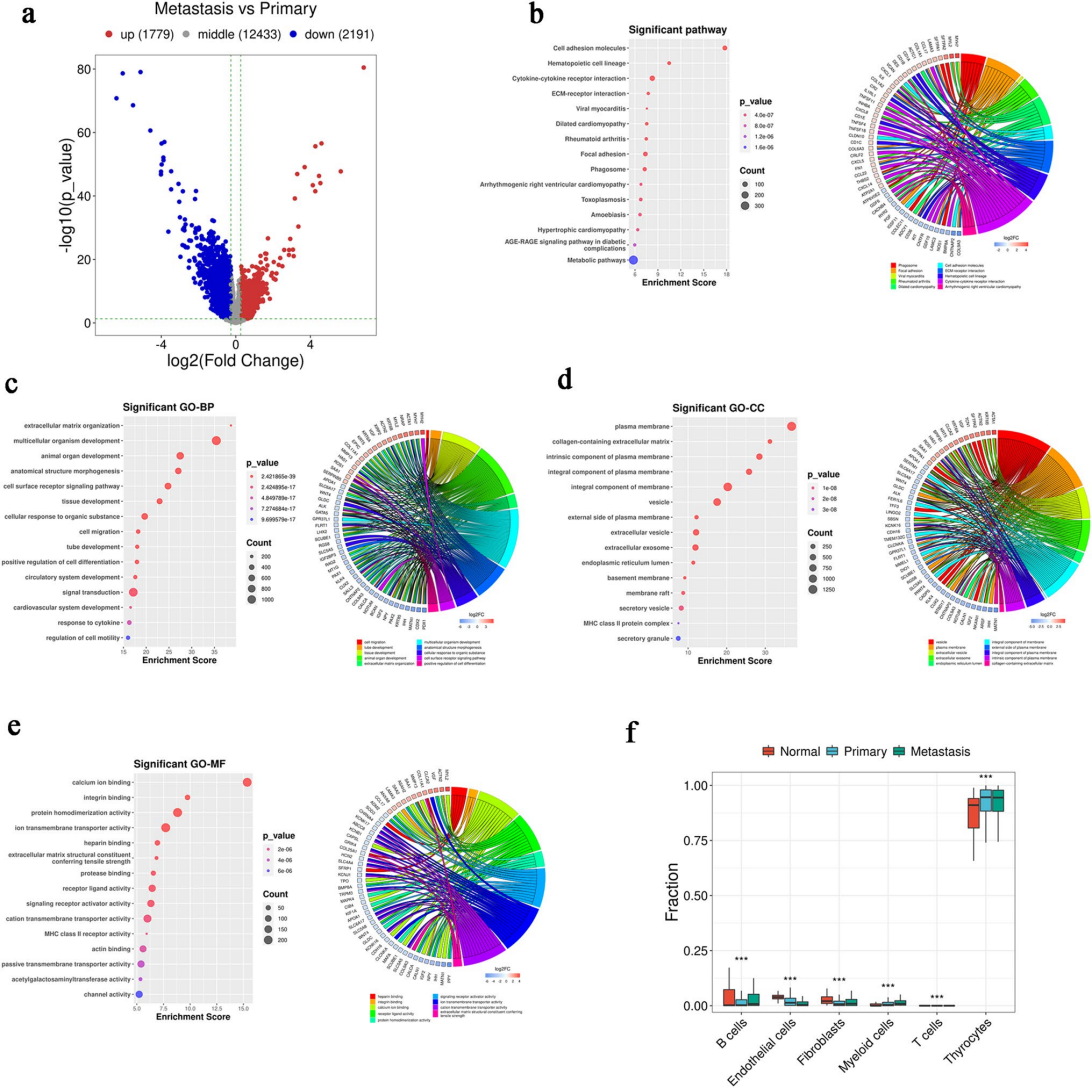
Heterogeneity of PTC LNM in malignant cells. **a** Volcano map of DEGs in malignant cell scRNA-seq data. **b** KEGG analysis of DEGs in scRNA-seq data and circle plot of the top ten enriched KEGG pathways corresponding to the first (maximum) 50 genes based on absolute log_2_FC values. **c**–**e** GO analysis of BP, CC, and MF for DEGs in scRNA-seq data and circle plot of the top ten enriched GO–BP, GO–CC, and GO–MF pathways corresponding to the first (maximum) 50 genes based on absolute log_2_FC values. **f** Cell–cell interaction network for malignant cell scRNA-seq data. **g** Top 50 TF activity differences between groups (absolute *t* value). **h** Expression and differences in TFs with an absolute *t* value > 25 between groups, ***p < 0.001

Based on the scRNA-seq data, malignant cells in thyrocytes were identified. A total of 271 upregulated and 406 downregulated DEGs were identified between the Metastasis and Primary groups (Fig. 2a). KEGG enrichment analysis of these DEGs, as summarized using a circle plot, revealed that the DEGs were significantly enriched in the autoimmune thyroid disease (AITD) pathway and were downregulated (Fig. 2b). GO enrichment analysis showed that the most enriched biological process, cell component, and molecular function were SRP-dependent co-translational protein targeting to the membrane, the cytosolic ribosome, and RNA binding, respectively (Fig. 2c–e). Cell–cell interaction network showed that malignant cells in PTC LNM were related to all listed cells, indicating that LNM is a complex biological process (Fig. 2f). Then, we investigated the regulatory activity of TFs in malignant cells, finding that EGR1 and YY1 were the two most distinct TFs (Fig. 3g, h).

### Functional differences between primary PTC and LNM

In addition to analyzing scRNA-seq data, we also divided bulk RNA-seq data into Metastasis (N1) and Primary (N0) groups for analysis. A total of 1779 upregulated DEGs and 2191 downregulated DEGs were identified from the bulk RNA-seq data (Fig. 3a). In KEGG pathway enrichment analysis, the DEGs were clearly enriched for cell adhesion molecules (Fig. 3b). GO enrichment analysis revealed that extracellular matrix organization and multicellular organism development were the most enriched biological processes (Fig. 3c), while the most enriched cellular component was the plasma membrane, suggesting that changes in the plasma membrane play an important role in PTC LNM (Fig. 3d). Meanwhile, calcium ion binding was the most enriched molecular function (Fig. 3e). Significant differences in the proportions of each cell type were identified between the Normal, Primary, and Metastasis groups (Fig. 3f).

### Identification of clinical diagnostic markers and construction of the LNM prediction model

**Fig. 4:**
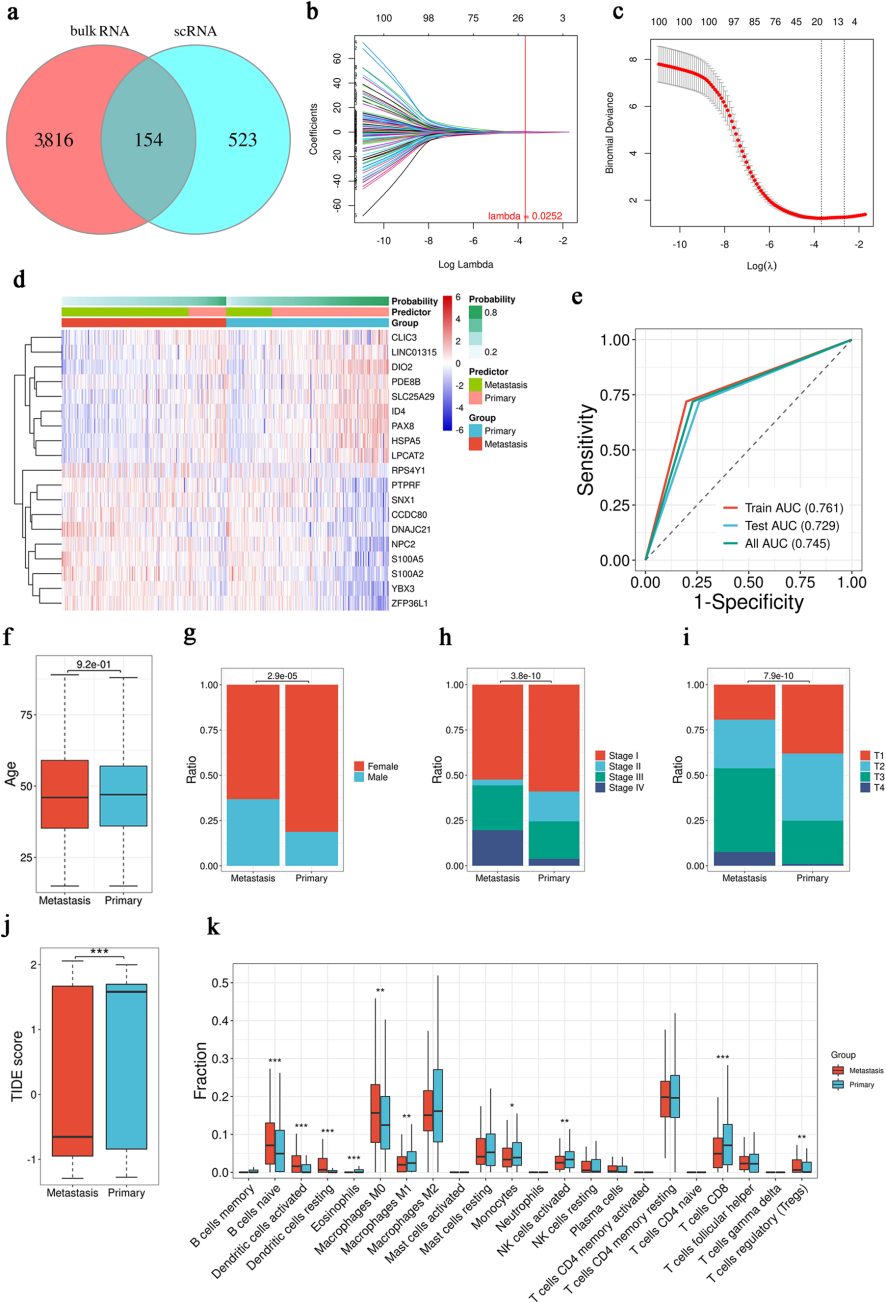
The selection of clinical diagnostic markers and the construction of PTC LNM prediction model. **a** Venn diagram of intersecting DEGs for scRNA-seq and bulk RNA-seq data. **b** Lasso regression screening for variable genes. **c** Cross validation. **d** Model tag gene (coefficient ≠ 0) expression cluster heatmap. **e** ROC for test, train, and all datasets. **f**–**i** Comparison of age, sex, clinical stage, and T stage between groups were divided by models. **j** Comparison of TIDE scores between groups were divided by models. **k** Difference in immune gene scores between groups, *p < 0.05, **p < 0.01, ***p < 0.001

There were 154 DEGs showed difference between Metastasis and Primary in both scRNA-seq and bulk RNA-seq (Fig. 4a). After genes with strong correlation had been removed, *S100A2*, *RPS4Y1*, *S100A5*, *ZFP36L1*, *CCDC80*, *YBX3*, *PTPRF*, *NPC2*, *DNAJC21*, and *SNX1* were screened as upregulated DEGs in the Metastasis group, while *LPCAT2*, *CLIC3*, *SLC25A29*, *PDE8B*, *PAX8*, *LINC01315*, *HSPA5*, *ID4*, and *DIO2* were screened as downregulated genes (Fig. 4b–d). Of these 19 genes, S100A2 was the most differentially upregulated gene and DIO2 was the most differentially downregulated gene. Half of all samples were then randomly selected as a training set to construct the diagnostic model and the other half were used as a validation set to verify its reliability. Validation showed that the AUC for the diagnostic model exceeded 0.7 (Fig. 4e). Although PTC LNM is affected by a lot of factors, our diagnostic model is still has a high accuracy. We then analyzed the clinical data of the patients grouped by the model. The results demonstrated that patients in the Metastasis group showed significant differences in sex, tumor stage, and clinical stage compared to those in the Primary group (*p* = 2.9e^−5^, *p* = 7.9e^−10^, *p* = 3.8e^−10^; Fig. 4f–i). Interestingly, the Primary group had significantly higher TIDE scores (Fig. 4j). At the same time, immune infiltration analysis showed significant changes in the infiltration of various immune cell types, especially CD8^+^ cytotoxic cells (*p* < 0.001, Fig. 4k), which suggested that immune escape mechanism plays an important role in PTC LNM.

### S100A2 and DIO2 are useful diagnostic markers in PTC LNM

**Fig. 5:**
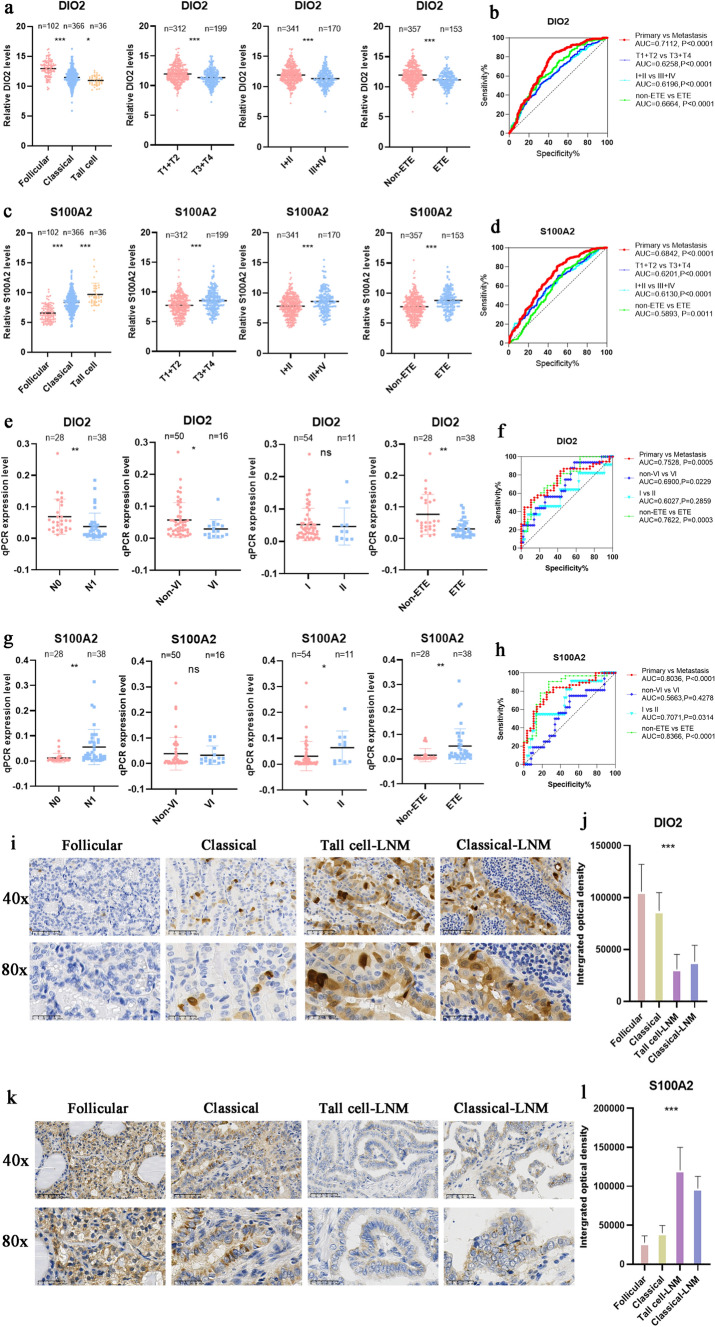
The verification of *S100A2* and *DIO2* using TCGA and tissue specimens. **a** Analysis of *DIO2* expression in patients with different clinicopathological characteristics in TCGA. **b** ROC curves for *DIO2* expression in patients with different clinicopathological characteristics in TCGA. **c** Analysis of *S100A2* expression in patients with different clinicopathological characteristics in TCGA. **d** ROC curves for *S100A2* expression in patients with different clinicopathological characteristics in TCGA. **e** RT-qPCR analysis of *DIO2* expression in 66 PTC specimens from patients with different clinicopathological characteristics. **f** ROC curves for *DIO2* expression in 66 PTC specimens from patients with different clinicopathological characteristics. **g** RT-qPCR analysis of *S100A2* expression in 66 PTC specimens from patients with different clinicopathological characteristics. **h** ROC curves for *S100A2* expression in 66 PTC specimens from patients with different clinicopathological characteristics. **i** IHC sections showing DIO2 expression in different PTC tissue subtypes at 40 × and 80 ×, respectively. **j** IHC quantitative analysis of DIO2 in different PTC subtypes. **k** IHC sections showing S100A2 expression in different PTC tissue subtypes at 40 × and 80 ×, respectively. **l** IHC quantitative analysis of S100A2 in different PTC subtypes. Data represent the mean ± standard deviation (SD), ^ns^*p* > 0.05, *p < 0.05, **p < 0.01, ***p < 0.001

Finally, we aimed to verify the effect of the two diagnostic markers genes with the highest amplification rate-*S100A2* and *DIO2-*in the Metastasis group. Analysis of the clinicopathological data for *DIO2* and *S100A2* in the TCGA database revealed significant differences in T stage, pathological type, clinical stage, and extrathyroid extension (ETE) between the two groups (*p* < 0.001; Fig. 5a–d). Therefore, we examined DIO2 and S100A2 RNA and protein expression in tissue samples from 66 patients with PTC (Fig. S1). RT-qPCR analysis verified the differences in both *DIO2* and *S100A2* expression between N0 and N1 samples (*p* < 0.01), while there was no significant difference in N1a and N1b (Fig. S1g, h). In terms of ETE, clear differences were shown in *DIO2* and *S100A2* expression between PTC tissues (*p* < 0.01). DIO2 differed in the presence or absence of vascular infiltration (VI), whereas S100A2 differed in clinical staging (*p* < 0.05; Fig. 5e–h). Meanwhile, IHC analysis showed that DIO2 expression was higher in samples with a follicular subtype than in those with a classical subtype and was lowest in the tall cell subtype (*p* < 0.001; Fig. 5i, j), whereas S100A2 expression showed the opposite trends (*p* < 0.001; Fig. 5k, l). The above results demonstrate the role of S100A2 and DIO2 in the clinical diagnosis of PTC.

### Roles of DIO2 in PTC LNM

**Fig. 6:**
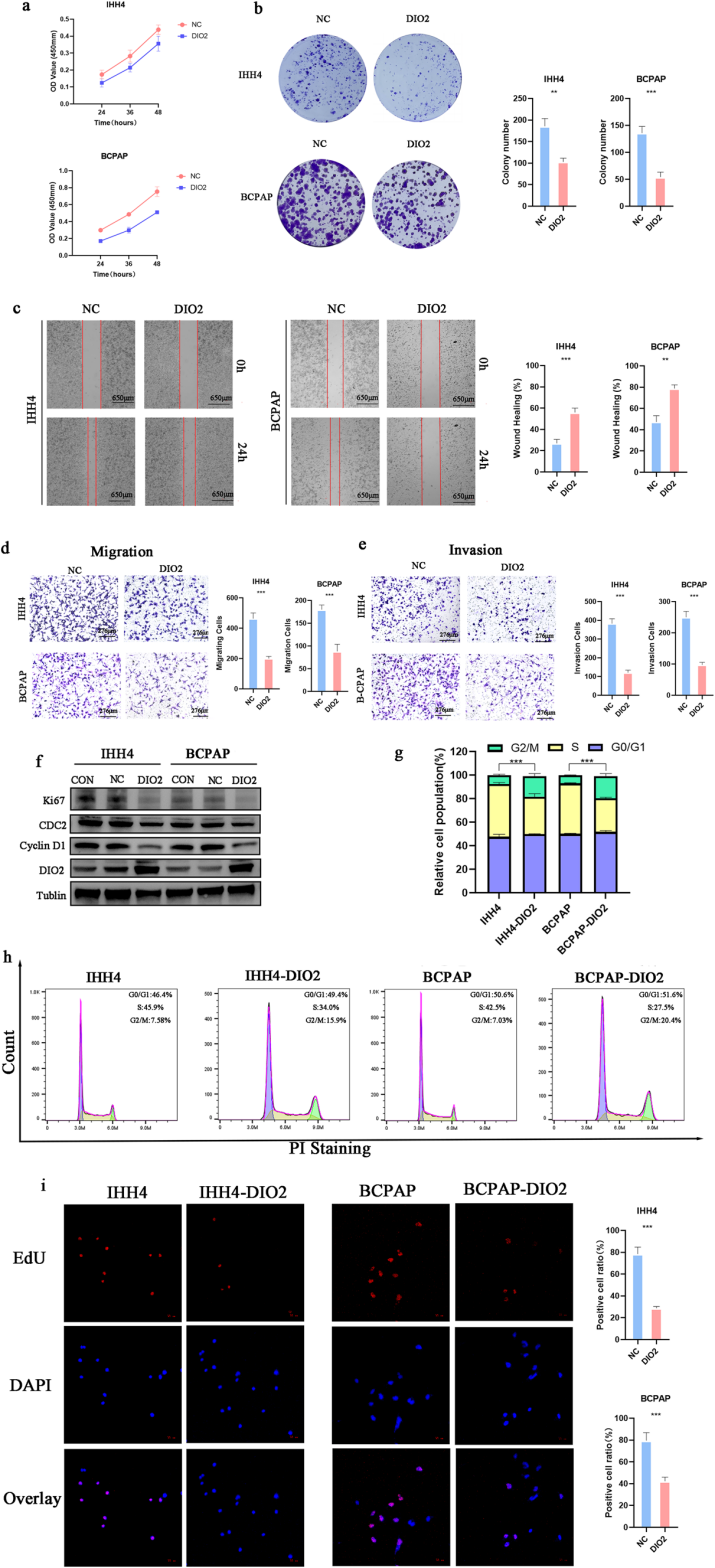
The biological validation of DIO2 in PTC cell lines. **a** Results of CCK-8 assay showing the viability of PTC cells after transfection with overexpressed DIO2 or NC. **b** Results of colony formation assay of PTC cells after transfection with overexpressed DIO2 or NC. **c** Results of wound-healing assay of PTC cells after transfection with overexpressed DIO2 or NC. Results of Transwell assays of PTC cell migration (**d**) and invasion (**e**) after transfection with overexpressed DIO2 or NC. **f** Protein levels of DIO2, Ki-67, cyclin D1, and CDC2 were measured by Western blot assays. **g** The proportion of cell population at each cell cycle phase relative to total phases. **h** Cell cycle phase distribution of DIO2 overexpression PTC cell line (IHH4 and BCPAP). **i** 5-Ethynyl-2-deoxyuridine incorporation assay of DIO2 overexpression PTC cell line (IHH4 and BCPAP). Data represent the mean ± SD, **p < 0.01, ***p < 0.001

Next, we investigated the relationship between *DIO2* and PTC by overexpressing DIO2 in PTC cells (BCPAP and IHH4). BCPAP and IHH4 cells that overexpressed DIO2 had a weak ability to clone and proliferate compared to NC (BCPAP: *p* < 0.001 and IHH4: *p* < 0.01, Fig. 6a, b) in colony formation assays (BCPAP: *p* < 0.01; and IHH4: *p* < 0.001, Fig. 6c) in wound-healing assays. Consistent findings were also observed for cell migration and invasion in both cell lines (*p* < 0.001; Fig. 6d, e), suggesting that DIO2 may play an inhibitory role in PTC progression.

**Fig.7:**
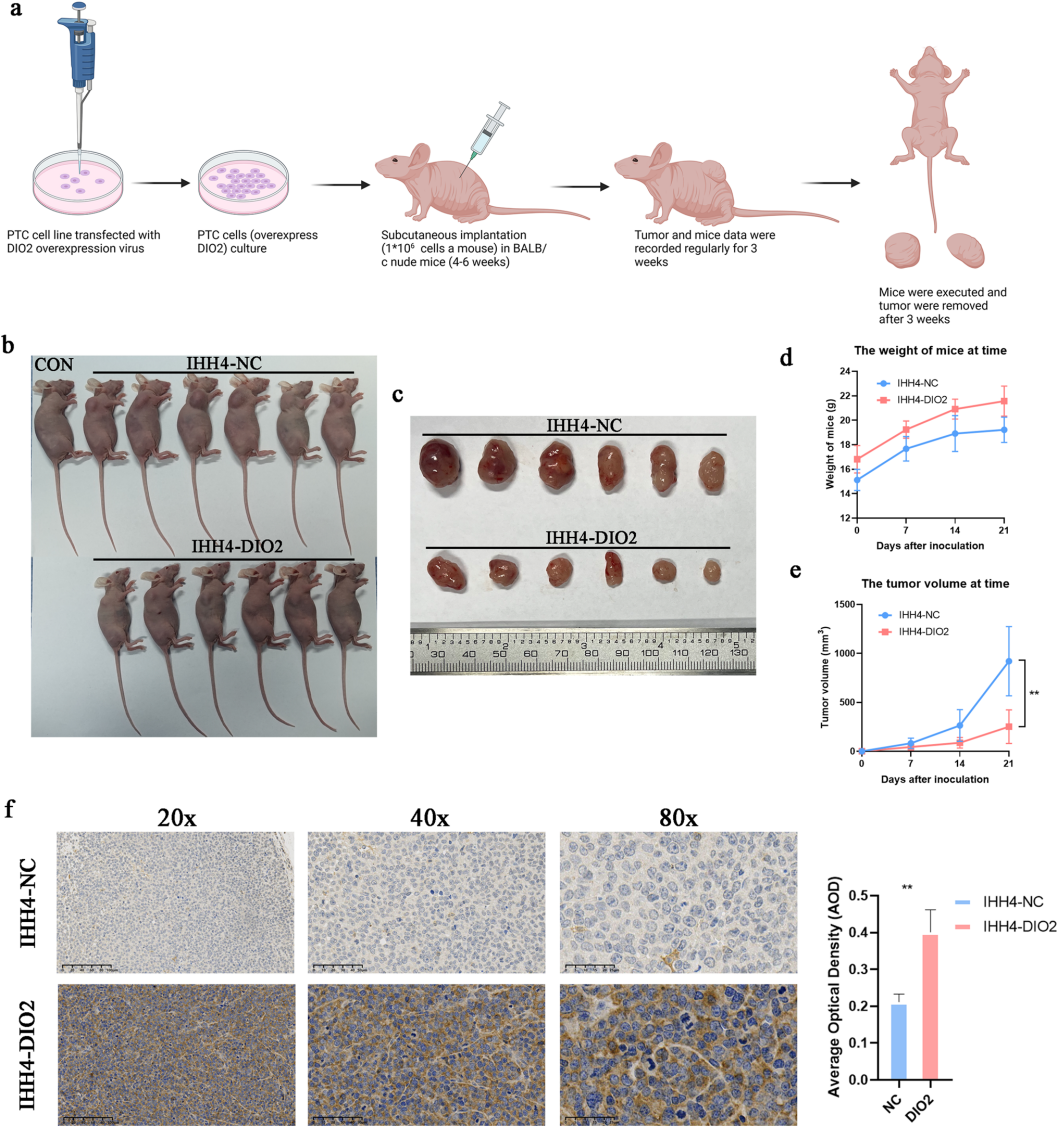
Validation of DIO2 in vivo experiments. **a** The schematic graph of xenograft studies. **b** Image of xenograft tumor mice. **c** Image of xenograft tumors. **d** Weight records of mice for 3 weeks in vivo. **e** Volume records of xenograft tumors (mm^3^). **j** IHC of xenograft tumors and their quantitative analysis at 20x, 40 ×, and 80 ×, respectively. Data represent the mean ± SD, **p < 0.01, ***p < 0.001

Further investigation of the mechanism of PTC inhibition made by DIO2. We found that overexpression of DIO2 decreased the expression of cell cycle proteins (Cyclin D1, CDC2) and proliferation protein (Ki-67) in PTC cells (Fig. 6f). The reason may be that PTC cells are more blocked in G2/M phase (*p* < 0.001, Fig. 6g, h). Cell cycle block affected the proliferation of PTC cells, and PTC cells overexpressed DIO2 in the EdU assay showed less fluorescence overlaps (*p* < 0.001, Fig. 6i). Further, we planted IHH4 overexpressed DIO2 in mice (Fig. 7a). The results showed that IHH4 grafted tumors overexpressed DIO2 were significantly smaller than those in the control group (*p* < 0.05, Fig. 7b–e). IHC analysis of the xenograft tumors showed that a large amount of overexpression of DIO2 was observed in the xenograft tumor cells. Under high magnification (80X), the stained area can be clearly seen to be located in the cytoplasm of the cell (*p* < 0.05; Fig. 7f).

## Discussion

Although PTC is considered to have a good prognosis, LNM of PTC remains the most concerning challenge for doctors and patients. In this study, we aimed to analyze LNM heterogeneity in malignant PTC cells by combining scRNA-seq and bulk RNA-seq data. From the screened diagnostic markers, we selected the two genes with the largest amplification for further study—*DIO2* and *S100A2*.

Unlike previous studies of S100A2 in PTC [31, 32], we found that S100A2 could have potential for the diagnosis of PTC LNM. Based on the previous literature, we hypothesize that S100A2 may affect LNM by suppressing immunity and regulating tumor glycolysis [11, 15]. In addition to being a diagnostic marker of PTC LNM, we found that S100A2 may be related to the malignancy of PTC, since S100A2 displays differential patterns of expression in different pathological types of PTC. Here, we found that DIO2 can be used as a diagnostic marker of LNM in PTC. Like S100A2, DIO2 is also associated with the malignant progression of PTC. Despite the clinical significance of these findings, we observed no significant differences in T staging or clinical staging with S100A2 and DIO2 expression. Therefore, future studies should validate our findings using a larger sample size.

When we analyzed the enriched pathways using the combined scRNA-seq and bulk RNA-seq data, we found that the AITD pathway intersected in both datasets. The relationship between AITD and PTC has been controversial, with some studies suggesting that AITD is a risk factor for PTC [33] and others suggesting that AITD is not directly related to PTC but is caused by an increase in TSH secretion due to AITD-induced hypothyroidism [34].

Since accurate LNM prediction in PTC is an important consideration when selecting treatments and determining patient prognosis, many studies have attempted to predict LNM from clinical data, imaging, and pathology [35–37]. However, these LNM diagnostic models have been based on tumor phenotypes, which can be affected by many factors. In comparison, the genome prediction model based on the origin of PTC LNM is more comprehensive and accurate. Moreover, unlike previous studies that only used bulk RNA-seq data for modeling, this study combined scRNA-seq data with bulk RNA-seq data [38], which can remove non-PTC cell interference from bulk RNA-seq data while allowing the model to be used at the tissue level, thereby avoiding the huge workload and cost associated with scRNA-seq while maintaining predictive accuracy and clinical feasibility. Although the early prediction of LNM for PTC treatment is undoubtably important, our model showed no difference in overall survival or disease-free survival between patient groups (Fig. S1a–f), likely due to the excellent prognosis of patients after PTC treatment. Therefore, it is important to find a characteristic measure of PTC prior to treatment.

Due to the large difference demonstrated by DIO2 in this study, we further explored its function in PTC LNM. Lymph-node metastasis plays a unique role in the treatment of PTC and dominates the selection of surgical scope. PTC cell proliferation was significantly inhibited, and they stayed more in G2/M phase when DIO2 was overexpressed. Its ability to migrate and invade is also affected. It is worth mentioning that the expression of DIO2 in anaplastic thyroid carcinoma has been found to promote the progression of tumors [39]; however, the authors acknowledge the different role of DIO2 in early and advanced thyroid cancer. The excellent prognosis of PTC also suggests that PTC is still a relatively early cancer even when lymph-node metastasis occurs. Therefore, we can still consider DIO2 as a potential target for controlling PTC progression.

## Conclusions

In this study, we explored the heterogeneity of LNM in PTC using scRNA-seq, thereby providing a reference for future research on LNM in PTC. By combining scRNA-seq and bulk RNA-seq data, we further constructed a PTC LNM diagnostic model and demonstrates its clinical applicability. On this basis, the effectiveness of S100A2 and DIO2 as diagnostic markers were further demonstrated. Further we found that DIO2 could inhibit PTC proliferation migration and invasion by blocking the PTC cell cycle, which has the potential to be a therapeutic target. Despite these important findings, this study also has some limitations. First, although the scRNA-seq data used in this study covered patients with different pathological types of PTC, the sample size was still small. Second, as a preliminary study, there are still many aspects of this study that require further elucidation. Finally, the diagnostic model established in this study requires more data for further validation. Given the high incidence of PTC, more scRNA-seq data from PTC are needed in the future to yield more concrete conclusions.

In summary, this study explored the heterogeneity of the PTC LNM and constructed a feasible LNM diagnostic model by combining scRNA-seq and bulk RNA-seq. Further, we validated the effectiveness of S100A2 and DIO2 as diagnostic markers and explored the mechanism of PTC inhibition by DIO2.

### Supplementary Information

Below is the link to the electronic supplementary material.

**Supplementary file 1. Fig. S1: a–d**: Survival analysis of overall survival, disease free survival and progress free survival in both groups. **e** Univariate and **f** multivariate Cox regression. **g **Analysis of *S100A2* and *DIO2* expression in patients with different N stages in TCGA. **h** Analysis of *S100A2* and *DIO2* expression in patients with different N stages in 66 PTC specimens from patients. (TIF 3203 KB)

## Data Availability

ScRNA-seq data (GSE184362) were downloaded from the Gene Expression Omnibus (https://www.ncbi.nlm.nih.gov/geo/query/acc.cgi). The Cancer Genome Atlas (TCGA)-Thyroid carcinoma (THCA) bulk RNA-seq and clinical data were downloaded from the Genomic Data Commons (GDC) data portal (https://portal.gdc.cancer.gov/).

## Abbreviations

PTC: Papillary thyroid carcinoma
LNM: Lymph-node metastasis
scRNA-seq: Single-cell RNA sequencing
RNA-seq: RNA sequencing
DEGs: Differentially expressed genes
DIO2: Type 2 deiodinase
RT-qPCR: Quantitative reverse transcription-polymerase chain reaction
IHC: Immunohistochemical
EdU: 5-Ethynyl-2′-deoxyuridine
KEGG: Kyoto encyclopedia of genes and genomes
GO: Gene ontology
TC: Thyroid carcinoma
PCA: Principal component analysis
UMAP: Uniform manifold approximation and projection
CNV: Copy number variation
TFs: Transcription factors
AUC: Under the curve
TIDE: The tumor immune dysfunction and exclusion
AITD: Autoimmune thyroid disease
ETE: Extrathyroid extension
VI: Vascular infiltration
