# Supplementary figures and images for: Single-cell and bulk RNA sequencing reveal heterogeneity and diagnostic markers in papillary thyroid carcinoma lymph-node metastasis
Source: J Endocrinol Invest. 2023 Dec 26;47(6):1513–30. doi: 10.1007/s40618-023-02262-6 (PMC11143037; doi:10.1007/s40618-023-02262-6)

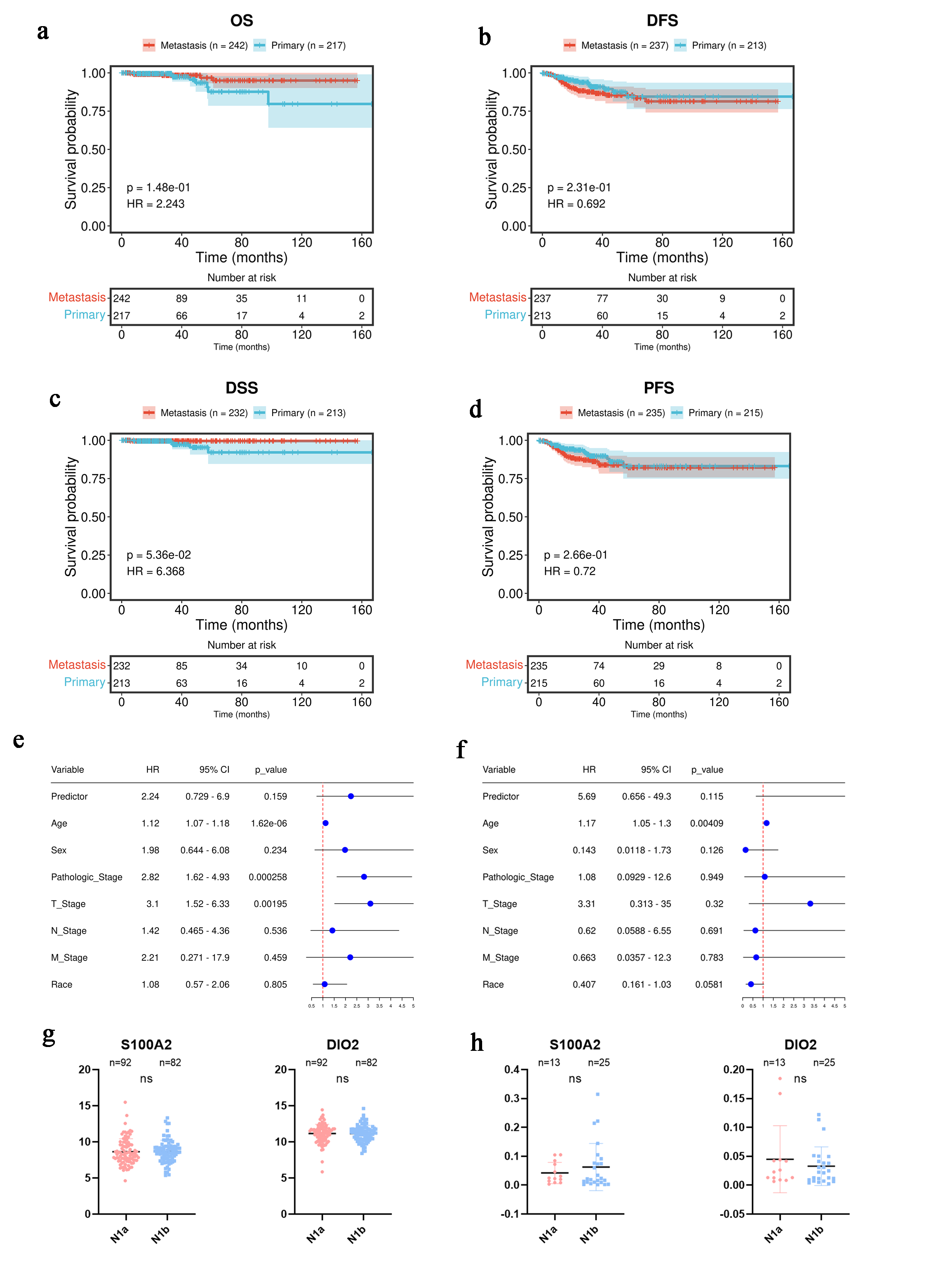

Supplement: Supplementary file 1 — Fig. S1: a–d: Survival analysis of overall survival, disease free survival and progress free survival in both groups. e Univariate and f multivariate Cox regression. g Analysis of S100A2 and DIO2 expression in patients with different N stages in TCGA. h Analysis of S100A2 and DIO2 expression in patients with different N stages in 66 PTC specimens from patients. (TIF 3203 KB) [file 40618_2023_2262_MOESM1_ESM.tif]
